# ICU-specific determinants of mortality in critically ill patients with infective endocarditis: Mortality predictors in ICU patients with infective endocarditis

**DOI:** 10.1186/s12879-026-13338-y

**Published:** 2026-05-19

**Authors:** Dilek Kuzukiran Kocatas, Behiye Deniz Kosovali, Nevzat Mehmet Mutlu, Gunal Bilek

**Affiliations:** 1 Department of Intensive Care Medicine, Ankara Bilkent City Hospital, Ankara, Türkiye Turkey; 2 Department of Business Administiration, İzmir Demokrasi University, İzmir, Türkiye Turkey

**Keywords:** Infective endocarditis, Intensive care unit, Mortality predictors, Fungal infective endocarditis, Critical illness, Septic embolism

## Abstract

**Objective:**

Infective endocarditis (IE) remains a life-threatening infectious disease characterized by high morbidity and mortality despite substantial advances in diagnostic and therapeutic strategies (Eur Heart J 44:3948–4042, 2023, Eur J Clin Microbiol Infect Dis 44:1325–1333, 2025). Patients requiring intensive care unit (ICU) admission represent the most severe clinical spectrum of IE and continue to exhibit markedly elevated mortality rates (Klimik Derg 32:2–116, Klimik Derg 36:216–225, 2023). Early identification of mortality predictors in critically ill patients is essential for optimizing multidisciplinary management strategies and improving clinical outcomes (Eur Heart J 44:3948–4042, 2023, Open Forum Infect Dis 12:ofaf628, 2025). The aim of this study was to evaluate clinical and laboratory predictors of mortality among patients with infective endocarditis managed in the intensive care unit of a tertiary care center.

**Methods:**

This retrospective cohort study included 59 adult patients with infective endocarditis who were managed in the intensive care unit of Ankara Bilkent City Hospital between 2022 and 2025. Demographic characteristics, comorbidities, microbiological findings, clinical severity scores, and laboratory parameters obtained at ICU admission were analyzed. The primary outcome was defined as in-ICU mortality. Receiver operating characteristic (ROC) curve analyses were performed to evaluate the predictive performance of the APACHE II score and the GCS. The AUCs of APACHE II and GCS were compared using DeLong’s test for correlated ROC curves. Independent predictors of mortality were identified using multivariable logistic regression analysis. Time-to-event analysis was conducted using a Cox proportional hazards regression model to evaluate factors associated with mortality during ICU follow-up. Internal validation of the logistic regression and Cox proportional hazards models was performed using bootstrap resampling (10000 iterations).

**Results:**

The mean age of the cohort was 62.3 ± 19.0 years. The overall in-ICU mortality rate was 52.5% (*n* = 31). ROC analysis demonstrated that the Glasgow Coma Scale showed a numerically higher discriminative performance compared with the APACHE II score (AUC: 0.758 vs. 0.717); however, the difference was not statistically significant. Multivariable logistic regression analysis identified lower GCS score (OR = 0.75, *p* = 0.017), presence of coronary artery disease (OR = 11.23, *p* = 0.008), fungal etiology (OR = 38.96, *p* = 0.008), and higher Nutritional Risk Screening (NRS) score (OR = 1.90, *p* = 0.043) as independent predictors of in-ICU mortality (Heart Fail Rev 30:1377–1395, 2025, Expert Consensus on the Prevention of Secondary Mucormycosis Following Respiratory Viral Infections, 2026). In Cox proportional hazards analysis, septic embolism (HR = 6.58, *p* = 0.047), coronary artery disease (HR = 3.23, *p* = 0.007), elevated total bilirubin (HR = 2.58, *p* = 0.006), increased creatinine (HR = 1.48, *p* = 0.006), elevated lactate dehydrogenase (HR = 1.001, *p* = 0.030), and lower albumin levels (HR = 0.92, *p* = 0.039) were independently associated with increased mortality hazard during ICU follow-up (Klimik Derg 32:2–116, 2020, Heart Fail Rev 30:1377–1395, 2025).

**Conclusion:**

Mortality among critically ill patients with infective endocarditis remains substantially high despite contemporary management strategies (Eur Heart J 44:3948–4042, 2023, Eur J Clin Microbiol Infect Dis 44:1325–1333, 2025). Neurological status assessed by GCS, nutritional risk evaluated by NRS, fungal etiology, and coronary artery disease emerged as key determinants of mortality risk, while septic embolism and markers of multiorgan dysfunction influenced mortality over time (Heart Fail Rev 30:1377–1395, 2025, Expert Consensus on the Prevention of Secondary Mucormycosis Following Respiratory Viral Infections, 2026). These findings highlight the importance of early risk stratification and multidisciplinary management and support the development of ICU-specific prognostic models to improve outcomes in this high-risk population (Eur Heart J 40:3222–3232, 2019, Open Forum Infect Dis 12:ofaf628, 2025).

**Clinical trial number:**

Not applicable.

## Introduction

Infective endocarditis (IE) is a critical infectious disease characterized by microbial invasion of the endocardial surface and cardiac valves and continues to be associated with high morbidity and mortality despite significant technological advances in diagnostic and therapeutic strategies [1, 2]. Globally, IE is considered the fourth most life-threatening infectious disease after sepsis, pneumonia, and intra-abdominal infections, and its clinical spectrum has undergone a notable epidemiological transformation in recent years [2, 3]. In developed countries, the mean age of patients with IE has increased to 60–75 years, while recent national data from Turkiye demonstrate a significant rise in median age to 57 years (*p* < 0.001), indicating a growing predominance of the disease in older populations [2, 4]. Although rheumatic heart disease has largely ceased to be a major predisposing factor in Western countries, sequelae of acute rheumatic fever remain responsible for approximately 37% of valve pathologies in Turkiye. However, the increasing use of intracardiac devices and healthcare-associated interventions has shifted the national epidemiological pattern toward that observed in developed countries [2, 5].

Regarding microbiological distribution, *Staphylococcus aureus* has emerged as the most frequently isolated pathogen both globally and in Turkiye [2, 6]. Large national cohort studies from Turkiye indicate that staphylococci (36.4%) are followed by streptococci (14.0%) and enterococci (11.9%) as the most common causative organisms, while *Brucella* spp. remains an endemic pathogen ranking fifth among etiological agents. In critically ill patients, particularly those exposed to invasive procedures and vascular catheterization, healthcare-associated IE cases present significant diagnostic and therapeutic challenges due to the predominance of resistant staphylococci and their fulminant clinical course [3, 7].

Patients with IE requiring intensive care unit (ICU) admission represent the most severe clinical spectrum of the disease. Previous studies have reported in-ICU mortality rates ranging between 45% and 68% among IE patients admitted to the ICU [8, 9]. Septic shock, acute heart failure, and neurological complications are consistently identified as major determinants of mortality in this population [1, 8]. In this critical setting, early diagnosis, prompt initiation of aggressive antimicrobial therapy, and timely surgical intervention guided by a multidisciplinary “endocarditis team” consisting of infectious disease specialists, cardiologists, and cardiovascular surgeons are essential for improving survival outcomes [1, 10].

However, despite advances in multidisciplinary management and modern antimicrobial strategies, data focusing specifically on intensive care unit populations remain limited, and ICU-specific prognostic determinants of mortality in infective endocarditis have not been fully elucidated.

In particular, the combined impact of neurological status, nutritional risk, and fungal etiology on mortality in critically ill patients with infective endocarditis has been insufficiently investigated in previous studies.

The present study aimed to analyze the epidemiological, microbiological, and clinical characteristics of 59 patients with infective endocarditis followed in the intensive care unit of Ankara Bilkent City Hospital, one of the largest tertiary referral centers in Turkiye, between 2022 and 2025. By identifying ICU-specific predictors of mortality, this study seeks to provide clinically relevant data to support early risk stratification and multidisciplinary management strategies in critically ill patients with infective endocarditis.

## Methods

### Data source and study population

This retrospective observational study was conducted using a structured dataset derived from the electronic medical records of 59 adult patients admitted to the intensive care unit (ICU) of Ankara Bilkent City Hospital between 2022 and 2025. All patients had complete demographic, clinical, laboratory, microbiological, and outcome data available for analysis. The diagnosis of infective endocarditis was established according to the modified Duke criteria, in accordance with the 2023 ESC Guidelines for the management of infective endocarditis. Only patients fulfilling definite or possible infective endocarditis criteria were included in the study.

Inclusion criteria were: age ≥ 18 years, diagnosis of infective endocarditis based on modified Duke criteria, and admission to the intensive care unit.

Exclusion criteria included: patients with incomplete clinical data, patients transferred from other centers with insufficient baseline information, and cases in which the diagnosis of infective endocarditis could not be confirmed.

The primary outcome of interest was defined as in-ICU mortality and recorded as a binary variable. Patients discharged alive from the ICU were considered survivors and censored at the time of ICU discharge. Deaths occurring after ICU discharge during the same hospital stay were not included in the primary endpoint. For time-to-event analyses, ICU length of stay (days) was used as the time variable, defined as the duration from ICU admission to death or ICU discharge.

### Variables

Demographic variables included age and sex. Comorbid conditions were recorded as binary variables and included hypertension, diabetes mellitus, heart failure, coronary artery disease (CAD), chronic obstructive pulmonary disease, chronic kidney disease, cerebrovascular disease, atrial fibrillation, and malignancy. Information on prior or concurrent valve surgery and valve replacement procedures (aortic, mitral, pulmonary, and tricuspid) was also collected.

Laboratory parameters obtained at ICU admission included hemoglobin, white blood cell count, neutrophil and lymphocyte counts, platelet count, neutrophil-to-lymphocyte ratio (NLR), urea, creatinine, liver function tests (AST, ALT, ALP, GGT), lactate dehydrogenase (LDH), total and direct bilirubin, albumin, C-reactive protein, and procalcitonin.

Clinical severity and physiological status were assessed using the Glasgow Coma Scale (GCS), Nutritional Risk Screening (NRS), and APACHE II score. ICU-related interventions included vasopressor use, mechanical ventilation, and duration of mechanical ventilation.

Infection-related variables comprised the presence of sepsis, septic embolism, causative microorganism (categorized as bacterial or fungal), diagnostic modality (transthoracic or transesophageal echocardiography), vegetation size, vegetation location, and valve type (native or prosthetic).

Fungal etiology was defined based on microbiological identification.

All patients received antimicrobial or antifungal therapy in accordance with current guideline recommendations and institutional protocols. Empirical antimicrobial therapy was initiated at ICU admission and subsequently adjusted according to microbiological culture results and antimicrobial susceptibility testing.

In cases of fungal infective endocarditis, targeted antifungal therapy was administered based on pathogen identification. The duration of antimicrobial or antifungal treatment was determined according to guideline recommendations and individualized based on clinical response and multidisciplinary team decisions.

### Statistical analysis

Continuous variables were examined for correlations using pairwise correlation analysis and visualized with a heatmap to assess potential multicollinearity. Correlation coefficients ≥ 0.80 were considered indicative of significant collinearity. When strong correlations were identified among biologically related variables, only one representative variable was retained for multivariable analyses to ensure model stability.

Receiver operating characteristic (ROC) curve analyses were performed to evaluate the discriminative performance of the APACHE II score and GCS in predicting in-ICU mortality. Areas under the ROC curve (AUCs) were calculated, and optimal cut-off values were determined using the Youden index. The AUCs of APACHE II and GCS were compared using DeLong’s test for correlated ROC curves.

Univariable logistic regression analyses were initially conducted to assess associations between candidate variables and in-ICU mortality. Variables with a p value < 0.10 were considered eligible for multivariable modeling. Composite severity scores were not included simultaneously with their individual components to avoid collinearity.

Multivariable logistic regression analysis was performed to identify independent predictors of in-ICU mortality. Model selection was guided by backward stepwise elimination based on the Akaike Information Criterion (AIC), with preference given to more parsimonious models when differences in AIC were < 2. Results were reported as odds ratios (ORs) with 95% confidence intervals (CIs). Model discrimination was evaluated using AUC values, and calibration was assessed graphically by comparing predicted and observed mortality probabilities.

To evaluate predictors of mortality over time, Cox proportional hazards regression analyses were conducted using ICU length of stay as the time scale and death as the event of interest. Patients discharged alive were treated as censored observations. Univariable Cox analyses were first performed, and variables with *p* < 0.10 were considered for multivariable modeling.

Variables demonstrating strong collinearity were excluded or consolidated before multivariable modeling. Mechanical ventilation variables were excluded due to near-complete separation with mortality, which would have resulted in unstable coefficient estimates. A multivariable Cox proportional hazards model was constructed using stepwise bidirectional elimination based on AIC minimization. Hazard ratios (HRs) with 95% CIs were reported.

Model performance was evaluated using the concordance index (C-index), and overall model significance was assessed using the likelihood ratio test. All statistical analyses were performed using R software, and a two-sided p value < 0.05 was considered statistically significant. No significant missing data were observed in the dataset; therefore, complete-case analysis was performed.

The number of variables included in multivariable models was limited in consideration of the events-per-variable principle.

Internal validation of the logistic regression and Cox proportional hazards models was performed using bootstrap resampling with 10,000 iterations. Model performance was evaluated using AUC and Harrell’s C-index, and confidence intervals were obtained using percentile-based bootstrap methods.

### Ethics statement

The study protocol was approved by the Institutional Ethics Committee of Ankara Bilkent City Hospital (Approval No: TABED 1-25-1406) and conducted in accordance with the ethical principles of the Declaration of Helsinki. Due to the retrospective design of the study, the requirement for informed consent was waived.

## Results

**Fig. 1:**
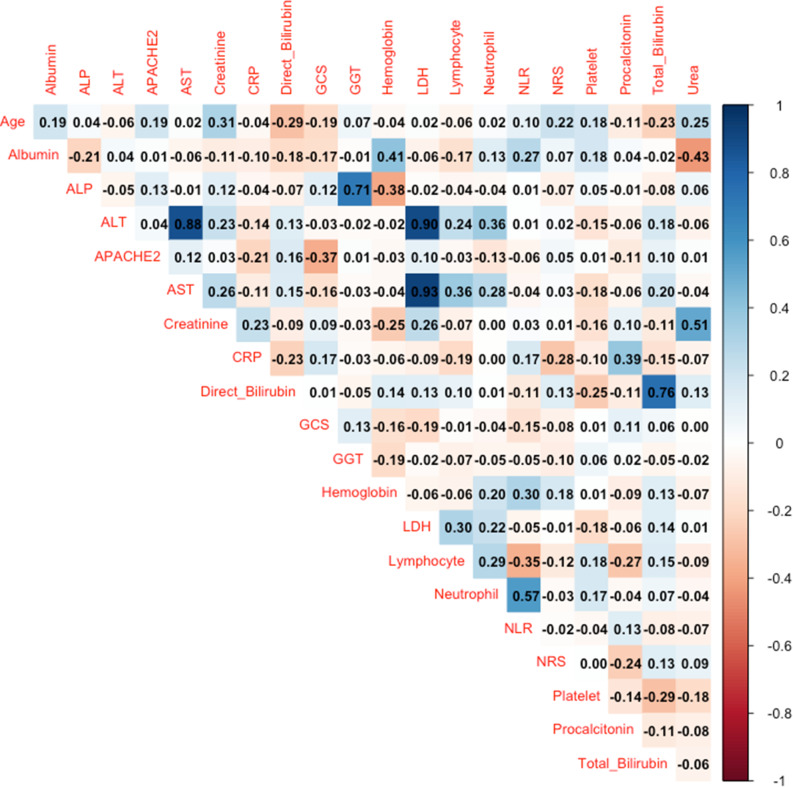
Heatmap showing pairwise correlations among demographic, clinical severity scores, and laboratory parameters

Figure 1 presents the heatmap of pairwise correlations among demographic variables, clinical severity scores, and laboratory parameters. Strong correlations were observed among liver-related biochemical markers, particularly between ALT and AST (*r* ≈ 0.88–0.93), and between transaminases and LDH (*r* ≈ 0.90). A high correlation was also noted between direct and total bilirubin (*r* ≈ 0.76). To avoid multicollinearity in subsequent analyses, highly correlated variables were not included simultaneously in multivariable models, and clinically representative markers were selected.

**Fig. 2:**
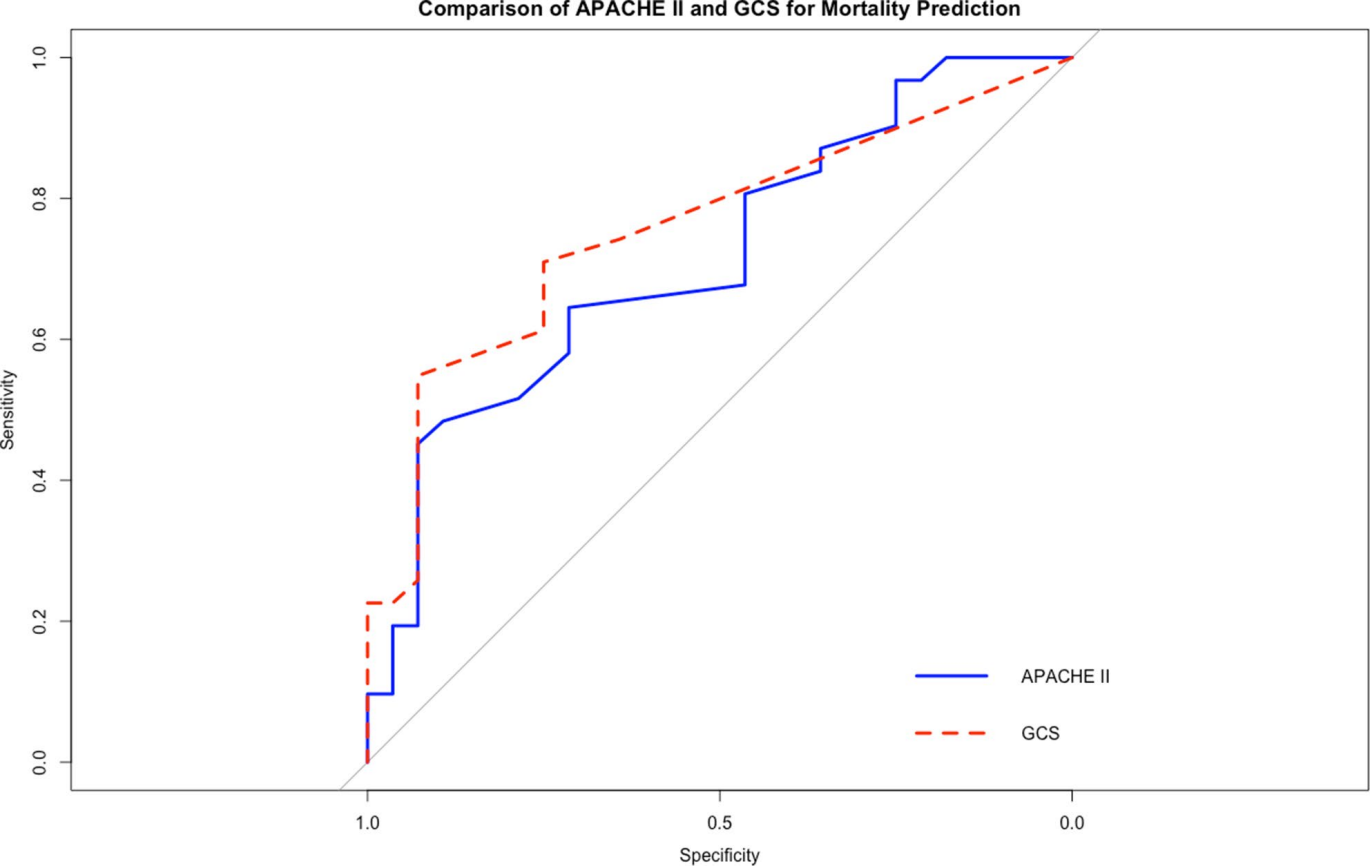
ROC curves of APACHE II and glasgow coma scale for predicting In-ICU mortality

ROC curve analysis demonstrated acceptable discrimination for both APACHE II and GCS in predicting in-ICU mortality (Fig. 2). Although GCS showed a numerically higher AUC than APACHE II (0.758 vs. 0.717), DeLong’s test indicated that the difference was not statistically significant (*p* = 0.589). The optimal cut-off value for APACHE II was 27.5, yielding a sensitivity of 45.2% and a specificity of 92.9%. For GCS, the optimal cut-off was 10.5, with a sensitivity of 54.8% and a specificity of 92.9%.

Univariable logistic regression analysis was performed to evaluate the association between candidate variables and in-ICU mortality. Variables meeting the predefined inclusion threshold (*p* < 0.10) were considered for multivariable analysis. These variables included mechanical ventilation (*p* < 0.001), Glasgow Coma Scale score (*p* = 0.002), APACHE II score (*p* = 0.008), duration of mechanical ventilation (*p* = 0.015), coronary artery disease (*p* = 0.020), causative microorganism (*p* = 0.028), valve type (*p* = 0.042), C-reactive protein level (*p* = 0.046), age (*p* = 0.068), ICU length of stay (*p* = 0.068), valve surgery (*p* = 0.074), vasopressor use (*p* = 0.075), tricuspid valve vegetation (*p* = 0.083), and nutritional risk score (*p* = 0.091).

The APACHE II score was not included in the multivariable model because of collinearity with the Glasgow Coma Scale and its lower discriminative performance. GCS was therefore retained for multivariable modeling. Mechanical ventilation was excluded due to near-complete separation with mortality, which would have resulted in unstable model estimates.

Following stepwise selection, two closely performing multivariable logistic regression models were identified. The first model included GCS, coronary artery disease, causative microorganism, tricuspid valve vegetation, and nutritional risk score, whereas the second, more parsimonious model excluded tricuspid valve vegetation. The more parsimonious model was preferred to reduce overfitting and improve stability. Although inclusion of tricuspid valve vegetation resulted in a slightly lower AIC (58.76 vs. 59.67), the difference was less than 2, indicating no meaningful improvement in model fit. Model discrimination remained excellent and comparable between models (AUC 0.90 vs. 0.89). Baseline characteristics of the study population stratified by survival status are presented in Table 1.

**Table 1:** Baseline characteristics of the study population stratified by in-ICU survival status

Variable	Survivors (*n* = 28)	Non-survivors (*n* = 31)	*p*-value
Age, years	61.00 [38.25–76.25]	68.00 [55.50–77.50]	0.131
GCS	15.00 [12.50–15.00]	10.00 [7.50–14.00]	< 0.001
APACHE II	21.00 [15.50–23.25]	26.00 [19.00–30.50]	0.004
NRS	5.00 [4.00–5.00]	5.00 [4.00–6.50]	0.111
Hemoglobin	9.05 [8.40–11.27]	9.30 [8.45–10.65]	0.933
WBC	11.99 [7.09–15.15]	9.00 [6.30–14.41]	0.400
Creatinine	1.87 [1.14–3.12]	2.13 [1.02–3.64]	0.826
Total bilirubin	0.63 [0.48–0.92]	0.86 [0.60–1.19]	0.111
Albumin	30.00 [26.50–32.25]	28.00 [24.00–31.50]	0.089
CRP	171.85 [122.58–225.20]	128.40 [57.55–175.40]	0.034
Procalcitonin	4.20 [0.85–11.15]	1.25 [0.27–7.44]	0.113
ICU length of stay, days	10.50 [8.50–30.00]	20.00 [10.00–38.50]	0.046
Mechanical ventilation duration, days	0.00 [0.00–0.25]	11.00 [3.00–33.50]	< 0.001
Vegetation size	2.00 [1.00–2.00]	2.00 [1.00–2.00]	0.334
Male sex, n (%)	14 (50.0)	14 (45.2)	0.797
Hypertension, n (%)	18 (64.3)	17 (54.8)	0.597
Diabetes mellitus, n (%)	12 (42.9)	16 (51.6)	0.604
Heart failure, n (%)	6 (21.4)	8 (25.8)	0.766
Coronary artery disease, n (%)	3 (10.7)	12 (38.7)	0.018
COPD, n (%)	3 (10.7)	6 (19.4)	0.477
Chronic kidney disease, n (%)	9 (32.1)	9 (29.0)	1.000
Cerebrovascular disease, n (%)	5 (17.9)	9 (29.0)	0.370
Atrial fibrillation, n (%)	2 (7.1)	5 (16.1)	0.428
Malignancy, n (%)	4 (14.3)	6 (19.4)	0.734
Valve surgery, n (%)	8 (28.6)	3 (9.7)	0.095
Vasopressor use, n (%)	8 (28.6)	16 (51.6)	0.111
Mechanical ventilation, n (%)	7 (25.0)	29 (93.5)	< 0.001
Septic embolism, n (%)	3 (10.7)	2 (6.5)	0.661
Sepsis, n (%)	19 (67.9)	19 (61.3)	0.786

In the multivariable logistic regression analysis, four variables were independently associated with in-ICU mortality (Table 2). Lower GCS scores were significantly associated with increased mortality (OR = 0.75 per 1-point increase, *p* = 0.017). The presence of coronary artery disease was associated with a markedly higher risk of mortality (OR = 11.23, *p* = 0.008), and fungal etiology was strongly associated with in-ICU mortality compared with bacterial infections (OR = 38.96, *p* = 0.008). Higher NRS values were also independently associated with increased mortality (OR = 1.90, *p* = 0.043).

**Table 2:** Multivariable logistic regression analysis for predictors of in-ICU mortality

Variable	β Coefficient	Std. Error	Odds Ratio (OR)	*p* value
Intercept	−0.600	2.382	0.55	0.801
GCS	−0.286	0.120	0.75	0.017
CAD (Yes)	2.418	0.916	11.23	0.008
Causative microorganism (Fungal)	3.662	1.378	38.96	0.008
Nutritional Risk Score (NRS)	0.643	0.318	1.90	0.043

AUC: 0.89, Null Deviance: 81.64, Residual Deviance: 49.67, AIC: 59.67

The final multivariable model demonstrated excellent discriminative performance, with an area under the receiver operating characteristic curve of 0.89. Internal validation using bootstrap resampling (10000 iterations) confirmed the robustness of the model, yielding a mean AUC of 0.902 (95% CI: 0.809–0.979). Also, a substantial reduction from null deviance (81.64) to residual deviance (49.67) indicated a significant improvement in model fit.

Fungal infective endocarditis was identified in 10 patients (10/59, 16.9%). Among these patients, mortality was notably high, with 9 deaths (90.0%). The identified fungal pathogens were predominantly Candida species, with occasional Aspergillus species.

All patients with fungal infective endocarditis received antifungal therapy in accordance with current guidelines. The most commonly used antifungal agents included echinocandins, amphotericin B, and azole-based therapies, and treatment was adjusted according to microbiological identification and clinical response.

Patients with fungal infective endocarditis frequently had underlying high-risk conditions, including central venous catheter use, immunosuppression, and other predisposing factors.

**Fig. 3:**
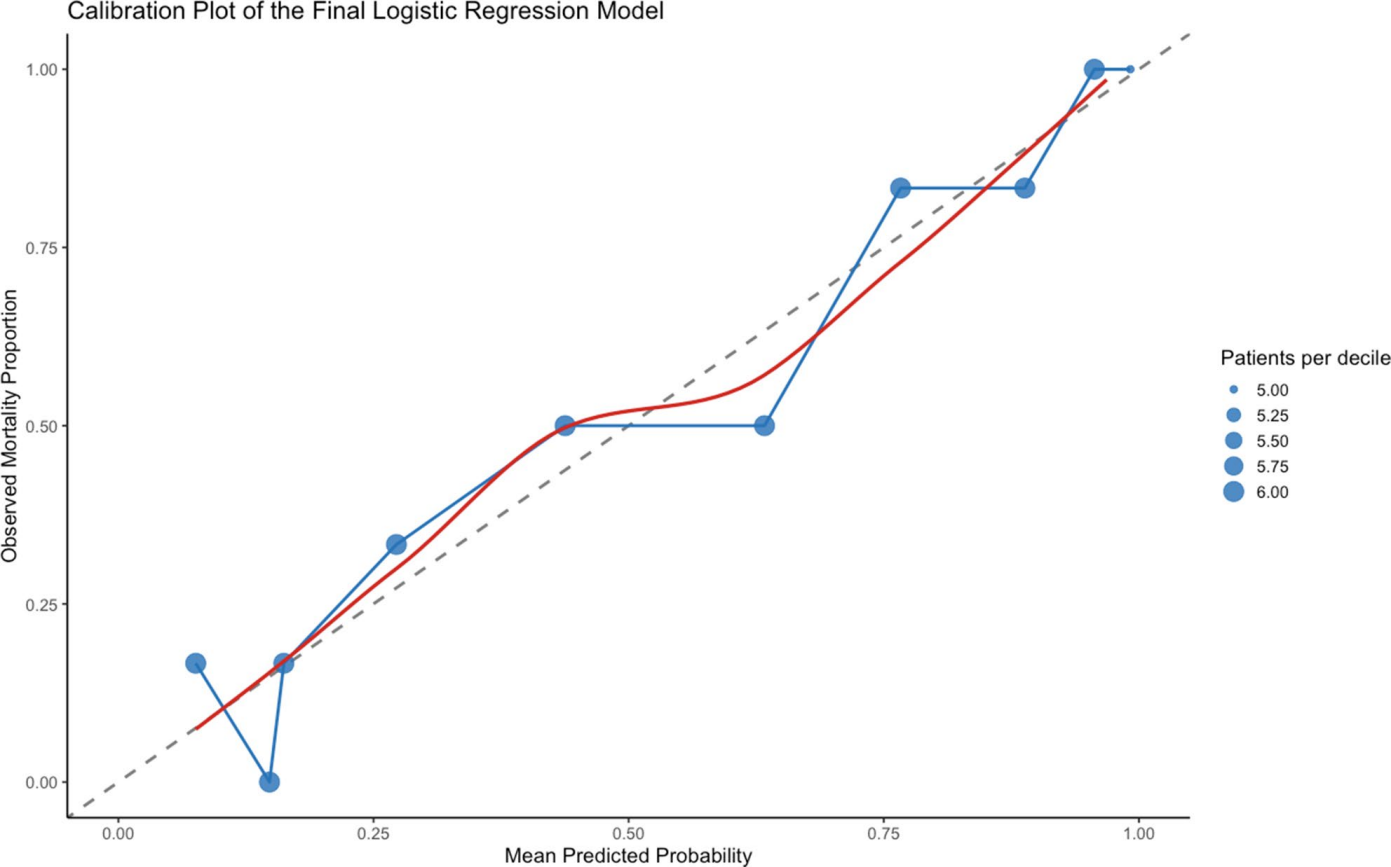
Calibration plot

Model calibration was assessed using a calibration plot comparing predicted and observed in-ICU mortality probabilities. The model demonstrated good calibration, with close agreement between predicted and observed mortality across the range of risk estimates. (Fig. 3)

**Fig. 4:**
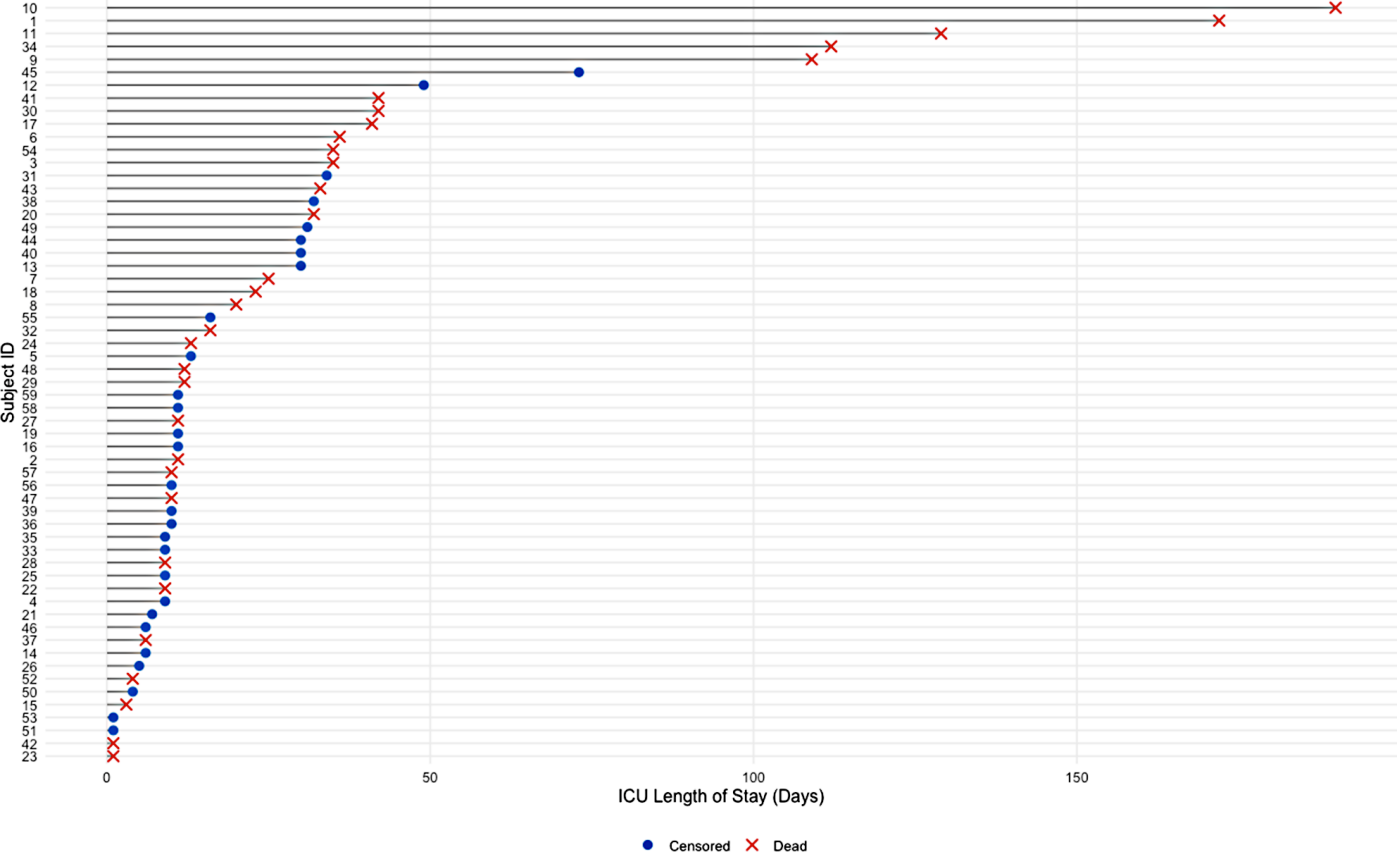
Swimmer plot showing time to death or ICU discharge for individual patients

Figure 4 presents a swimmer plot illustrating ICU length of stay and survival outcomes for individual patients. Each horizontal line represents a single patient, with line length corresponding to ICU duration. Deaths are indicated by crosses, whereas patients discharged alive are shown as right-censored observations. Substantial heterogeneity in ICU length of stay was observed, and the presence of censored observations supported the use of a time-to-event analytical approach.

To identify predictors of mortality over time, univariable Cox proportional hazards analyses were performed. Variables demonstrating a potential association with mortality at a significance threshold of *p* < 0.10 were considered for inclusion in the multivariable model. Based on univariable analyses, the following variables met the inclusion criterion: ALT (*p* < 0.001), vasopressor use (*p* = 0.002), creatinine (*p* = 0.003), duration of mechanical ventilation (*p* = 0.003), coronary artery disease (*p* = 0.005), AST (*p* = 0.017), urea (*p* = 0.025), LDH (*p* = 0.027), albumin (*p* = 0.028), tricuspid valve vegetation (*p* = 0.036), total bilirubin (*p* = 0.049), mechanical ventilation (*p* = 0.050), septic embolism (*p* = 0.064), sepsis (*p* = 0.077), and hemoglobin (*p* = 0.088).

Prior to multivariable modeling, variables demonstrating strong collinearity or methodological limitations were excluded to ensure model stability. Among correlated liver-related biomarkers, LDH was retained as a representative marker. Mechanical ventilation–related variables were excluded due to near-complete separation with mortality, and APACHE II was not included to avoid redundancy with other severity indicators.

A multivariable Cox proportional hazards model was constructed using stepwise bidirectional elimination based on minimization of the Akaike Information Criterion (AIC) to identify independent predictors of mortality and achieve a parsimonious model with optimal fit.

**Table 3:** Multivariable cox proportional hazards model for predictors of in-ICU mortality hazard

Variables	Hazard Ratio (HR)	95% Confidence Interval (CI)	*p*
Septic Embolism (Yes)	6.58	1.02–42.27	0.047*
CAD (Yes)	3.23	1.38–7.58	0.007*
Total Bilirubin	2.58	1.32–5.04	0.006*
Creatinine	1.48	1.12–1.97	0.006*
LDH	1.001	1.000–1.001	0.030*
Albumin	0.92	0.85–0.99	0.039*

Model Statistics: Concordance Index (C-index): 0.813 (se = 0.05); Likelihood ratio test: $\:{\boldsymbol{\chi\:}}^{2}$ = 42.65, *p* < 0.001

The results of the multivariable Cox proportional hazards regression analysis with ICU length of stay as the time scale and death as the event of interest are presented in Table 3. Septic embolism was independently associated with an increased mortality hazard (HR = 6.58, 95% CI: 1.02–42.27, *p* = 0.047). Coronary artery disease was also identified as an independent predictor of mortality (HR = 3.23, 95% CI: 1.38–7.58, *p* = 0.007). Higher total bilirubin levels (HR = 2.58, 95% CI: 1.32–5.04, *p* = 0.006), elevated creatinine (HR = 1.48, 95% CI: 1.12–1.97, *p* = 0.006), and increased LDH (HR = 1.001, 95% CI: 1.000–1.001, *p* = 0.030) were associated with increased mortality hazard. In contrast, higher albumin levels were independently associated with reduced mortality hazard (HR = 0.92, 95% CI: 0.85–0.99, *p* = 0.039).

The final model demonstrated good discriminative performance, with a concordance index (C-index) of 0.813 (SE = 0.05). Internal validation using bootstrap resampling (10000 iterations) yielded a mean C-index of 0.833 (95% CI: 0.724–0.927), supporting the stability of the Cox model. Overall model fit was statistically significant according to the likelihood ratio test (χ² = 42.65, *p* < 0.001).

## Discussion

This study demonstrates that patients with infective endocarditis (IE) requiring intensive care unit (ICU) admission represent the most severe clinical spectrum of the disease and continue to experience high mortality despite advances in diagnostic and therapeutic strategies [1–3]. Previous studies have reported in-ICU mortality rates ranging from 45% to 68% among critically ill patients with IE [3, 4]. The findings of the present cohort provide additional insight into ICU-specific prognostic factors by identifying neurological status, nutritional risk, fungal etiology, and coronary artery disease as major determinants of mortality. Unlike previous studies focusing primarily on general infective endocarditis populations, this study specifically evaluates critically ill ICU patients and simultaneously examines neurological status, nutritional risk, and fungal etiology within a unified prognostic framework.

Coronary artery disease (CAD) emerged as a strong and consistent predictor of mortality across both logistic and Cox regression models, indicating that cardiovascular comorbidity substantially contributes to adverse outcomes by increasing both baseline vulnerability and the rate of clinical deterioration during ICU stay.

The two statistical models highlighted complementary dimensions of mortality risk. Logistic regression identified factors associated with the overall probability of death, including neurological status, nutritional risk, and fungal etiology, whereas the Cox proportional hazards model emphasized time-dependent predictors reflecting systemic organ dysfunction and metabolic stress, such as septic embolism, renal dysfunction, hyperbilirubinemia, LDH elevation, and hypoalbuminemia. These findings suggest that certain variables influence the likelihood of death, while others determine the pace at which mortality risk progresses during ICU follow-up.

Neurological status assessed by the GCS demonstrated a numerically higher AUC than the APACHE II score in predicting in-ICU mortality; however, this difference was not statistically significant. Neurological complications, including septic embolism and cerebrovascular events, are well-established predictors of poor prognosis in IE and have been reported in 20–55% of cases [6–8]. The association between lower GCS scores and increased mortality underscores the importance of systematic neurological evaluation alongside conventional severity scoring in critically ill IE patients.

Fungal etiology was identified as a strong predictor of mortality in our cohort; however, this finding should be interpreted with caution given the relatively small number of fungal cases (*n* = 10), which may have led to an overestimation of the effect size in the multivariable model.

Nevertheless, this observation is clinically plausible. Candida species are among the leading causes of invasive fungal bloodstream infections and are associated with high mortality rates, particularly in critically ill patients with central venous catheters, prolonged hospitalization, broad-spectrum antibiotic exposure, and immunosuppression. Recent studies have emphasized the importance of early diagnosis, prompt initiation of antifungal therapy, and species-specific management strategies in improving outcomes in candidemia and related invasive infections [11].

Similarly, Aspergillus infections represent a significant cause of severe invasive disease in critically ill and immunocompromised patients. Their angioinvasive nature and often delayed diagnosis contribute to poor prognosis and increased mortality [12].

Although Mucorales species were not identified in our cohort, recent expert consensus highlights the importance of recognizing secondary mucormycosis in high-risk patients, particularly in the context of immune dysregulation and severe systemic illness [13].

Therefore, while fungal infective endocarditis appears to be an important prognostic factor, the magnitude of its effect should be interpreted cautiously, and larger multicenter studies are needed to better define the impact of specific fungal pathogens in ICU populations. The timing of surgical intervention remains a critical component of IE management. In our cohort, indications for early surgery and heart failure complications were consistent with urgent and emergent surgical criteria defined in the 2023 ESC guidelines [14, 15]. However, the high operative risk in patients with septic shock necessitates individualized decision-making within a multidisciplinary “endocarditis team” framework integrating infectious disease, cardiology, and cardiovascular surgery expertise [2, 16, 17].

Overall, these findings emphasize the importance of early risk stratification and comprehensive multidisciplinary management in critically ill patients with IE. Identification of ICU-specific prognostic factors may facilitate timely clinical decision-making and support the development of tailored risk prediction models for this high-risk population.

Despite the relatively limited sample size and the absence of a priori sample size calculation, internal validation using bootstrap resampling was performed to enhance the robustness and reliability of the findings. Given the relatively small number of events in our cohort, the number of variables included in multivariable models should be interpreted in the context of the events-per-variable principle. Although model selection was performed to achieve parsimony and internal validation was conducted using bootstrap resampling, the risk of overfitting cannot be completely excluded. The consistency of results across both logistic and Cox regression models further supports the clinical relevance of the identified predictors. However, several limitations should be acknowledged. First, the retrospective single-center design and modest sample size may limit the generalizability of the findings. In addition, only one patient in the cohort underwent surgical intervention, precluding meaningful statistical evaluation of the impact of surgical timing and postoperative outcomes on mortality. This distribution likely reflects the high severity of illness and surgical ineligibility of critically ill ICU patients rather than institutional treatment bias. Accordingly, these findings represent real-world characteristics of severe infective endocarditis in the intensive care unit, where many patients are not suitable surgical candidates because of advanced comorbidities and clinical instability. Furthermore, treatment strategies and surgical decision-making were not fully standardized and may have influenced outcomes. Nevertheless, the use of detailed ICU-based clinical data and robust multivariable statistical modeling provides clinically meaningful insight into mortality predictors in this high-risk population.

## Conclusion

Data obtained from the intensive care unit of Ankara Bilkent City Hospital demonstrate that infective endocarditis continues to retain its status as a “critical infection” associated with high mortality even in the era of modern medicine. Our findings indicate that neurological status assessed by the GCS and nutritional risk evaluated by the NRS may provide additional prognostic information beyond conventional severity scoring systems such as APACHE II.

Notably, the coexistence of fungal etiology and neurological deterioration was associated with markedly increased predicted mortality in this critically ill population. To improve survival in such high-risk patients, prompt initiation of appropriate antimicrobial therapy and timely surgical intervention in accordance with ESC guideline recommendations should be considered within a specialized multidisciplinary “Endocarditis Team” framework.

Future strategies should focus on the development of national risk stratification models incorporating ICU-specific prognostic factors and on the broader implementation of multidisciplinary management protocols tailored to critically ill patients with infective endocarditis.

Integration of neurological, nutritional, and microbiological parameters into ICU-specific risk assessment may improve early clinical decision-making and guide individualized therapeutic strategies in patients with infective endocarditis.

## Data Availability

The datasets used and/or analyzed during the current study are available from the corresponding author on reasonable request.

## Abbreviations

IE: Infective endocarditis
ICU: Intensive care unit
GCS: Glasgow Coma Scale
NRS: Nutritional Risk Screening
CAD: Coronary artery disease
ROC: Receiver operating characteristic
AUC: Area under the curve
LDH: Lactate dehydrogenase
